# Isotopic analysis of formula milk reveals potential challenges in geolocating bottle-fed babies

**DOI:** 10.1038/s41598-024-54173-y

**Published:** 2024-02-13

**Authors:** Lisette M. Kootker, Saskia T. M. Ammer, Gareth R. Davies, Christine Lehn

**Affiliations:** 1 Department of Earth Sciences, Geology & Geochemistry Cluster, Faculty of Science, Vrije Universiteit Amsterdam, de Boelelaan 1085, 1081 HV Amsterdam, the Netherlands; 2 Co van Ledden Hulsebosch Center (CLHC), Science Park 904, 1098 XH Amsterdam, the Netherlands; 3 Department of Forensic Medicine, Ludwig-Maximilians-Universität München, Nußbaumstraße 26, 80336 Munich, Germany

**Keywords:** Geochemistry, Biogeochemistry

## Abstract

In forensic investigations involving the identification of unknown deceased individuals, isotope analysis can provide valuable provenance information. This is especially pertinent when primary identifiers (i.e., DNA, dactyloscopy, etc.) fail to yield matches. The isotopic composition of human tissues is linked to that of the food consumed, potentially allowing the identification of regions of origin. However, the isotopic composition of deceased newborns and infants fed with milk formula may be influenced by that of the prepared milk. The findings contribute towards the possibility to isotopically identify bottle-fed infants. More importantly, the data convincingly show that the Sr isotope composition of the prepared milk is determined by that of the formula and not the (local) tap water, thereby limiting the potential of Sr isotope analysis for determining the geological or geographical origin in formula-fed babies in medico-legal cases.

## Introduction

Infanticide and neonaticide, complex and distressing phenomena that are often influenced by a combination of social, cultural, economic, and psychological factors, are historically documented in various cultures and societies^1^. Although intentional killing of children is illegal, some countries, including the Netherlands, have seen a fluctuating but slightly upward trend in infanticide or neonaticide cases^2,3^. Based on 2020 data from the WHO Mortality Database, between 0.3 (United Kingdom) and 5.6 (Latvia) children under 1 year were murdered per 100,000 inhabitants in Europe, slightly less than in the United States (6.2 per 100,000 inhabitants)^4^. In the Netherlands, the death rate fluctuates between 0.6 in 2013 and 2018 (N = 1), and 4.2 in 2020 (N = 7). Eleven cases (1.4 per 100,000 inhabitants) were reported in Germany in 2020, showing a significant decline since 2001 (N = 28, 3.7 per 100,000 inhabitants).

In the context of medicolegal cases, the remains of deceased infants in different stages of decomposition are occasionally discovered. These cases are not limited to instances of intentional homicide involving young children but may also encompass the (illegal) disposal of stillborn infants. Without any indications of the biological mother, a positive identification of the deceased is often impossible. However, recent developments in DNA research allow the suspected biological mother to be apprehended and detained years after the incident, as demonstrated in the Netherlands in 2022 where a woman was suspected of infanticide and the disposal of the boy's body 15 years earlier in 2006 ('Sem Vijverberg'), after the cold case was brought to the public's attention in 2021. Unfortunately, there are many cases where positive identification through primary identifiers or DNA is impossible. In such instances, isotopic analysis can provide new information to aid forensic investigations.

Several radiogenic and stable isotope systems, *inter alia* strontium (^87^Sr/^86^Sr), lead (^20x^Pb^/20x^Pb), oxygen (δ^18^O), nitrogen (δ^15^N), hydrogen (δ^2^H), carbon (δ^13^C), and sulphur (δ^34^S), provide valuable information about human diet and geographical provenance^5–14^. Isotopic research on human or faunal remains is based on the premise ‘you are (isotopically) what you eat’. This principle is related to the fact that these elements are incorporated into the human body through food and drinking water^15^. Isotopic data on keratin, collagen, and bioapatite are interpreted by comparing the human isotope data with the 'local' isotopic signatures of the environment, allowing to determine whether the human isotopic data obtained are compatible with a certain country or region using databases containing relevant reference isotopic data or relevant predictive maps of the isotopic landscape (‘isoscape’)^16–19^.

Since 2014, a collaborative effort involving Vrije Universiteit Amsterdam, the Netherlands Forensic Institute (NFI), and Ludwig-Maximilians-Universität München has been conducted to perform multi-isotopic analysis on 12 forensic cases involving the skeletonised remains of newborns, presumed to be either stillborn or killed shortly after birth, from the Netherlands and Germany. The primary objective of these investigations was to gain insight into the geographical origin of their birth mother. An optimal approach to ascertain the geographic origin and circumstances of the mother of the child involves the collection of multiple tissue samples, including hair or nails, ribs, and femurs of the newborn. These samples were analysed using stable C-N-S-H isotope and radiogenic Sr-Pb isotope analysis, allowing valuable insights into the provenance of the mother and child during and possibly shortly after pregnancy due to the formation of these tissues at various stages of life.

For these newborns, bone formation and growth and hair growth occurred during pregnancy. The development of the scalp hair follicles of the foetus begins around 3 to 5 months in utero, and hair growth starts around the 6th month of pregnancy, with the highest growth rate in the last trimester, though exact foetal hair growth rates are unknown^20–22^. Therefore, the hair tips carry information from an earlier stage of pregnancy compared to the proximal hair section. The bones contain the isotopic information of approximately the last 3–4 weeks of life due to their rapid turnover rates during foetus growth^7^. The Sr-C-N-S-H isotope compositions therefore reflects the mother’s diet during pregnancy, albeit that C-N-S-H undergo isotopic fractionation controlled by metabolically processes, potentially offering information on the geographical origin of the mother. The incorporation mechanisms for lead (Pb), a toxic metal, are more intricate. Lead accumulates in the body, predominantly stored in bones. During pregnancy and the postnatal period, Pb is mobilised from the mother’s bones and released back into the bloodstream, representing the dominant source of exposure to the developing foetus^23^. Consequently, the Pb found in foetal and breastfed newborn tissues reflects the mother’s Pb that could have been consumed several years before pregnancy. Thus, Pb isotope ratios serve as indicators of mother's origin before and possibly during pregnancy.

Determining the precise age at death for skeletonised newborns poses challenges. If they survived for several days or weeks, they must have been breastfed or milk formula fed. The bone turnover rate during the first months after birth is high, due to the rapid and dynamic growth in this early stage of life. For example, from the fifth to the 30th day after birth, the child's bone mass increases on average by 25%, and the annual remodelling rate of the bone material is c. 300% in the first year of life^24^. However, precise percentages of bone growth during this period remain elusive, primarily due to the multifaceted nature of contributing factors, including maternal health during pregnancy (e.g., smoking, alcohol or drug abuse), gestational age, birth weight, among others. Nevertheless, the swift turn-over rate implies a very rapid incorporation and expansion of nutritional components into the bone tissues and a complete replacement of the "old" bone material from the foetal phase of life with "new" material from the postnatally ingested food. If the child is fully breastfed after delivery, it continues to receive elements from a maternal source. If the child is instead fed industrial infant formula, the isotopic signatures in growing infant body tissues match the isotopic signature of the postnatally fed infant formula, presumably after a few weeks. Significant intra-individual tissue variabilities in the newborn may result from changes in the mother's diet during pregnancy, potentially indicating a change of residence. However, if the newborn lived for several days or weeks after birth, the observed shifts in isotopic signatures between hair and bone and between different skeletal elements (e.g., rib-femur) could also be due to the isotopic composition of the milk formula used.

At birth, the hair *δ*^13^C values of newborns are strongly associated with the isotopic signature of maternal hair, whereas the *δ*^15^N values are enriched by about 1‰^25^. The supply of breast milk leads to a further increase in hair *δ*^13^C and *δ*^15^N of 1–2‰ and 2–3‰ respectively in the neonatal body tissues compared to that of the mother^26,27^. In addition, a notable difference of c. 5‰ for *δ*^13^C and 4‰ for *δ*^15^N is observed between maternal milk and the infant’s tissues^28^. In contrast, the Sr-Pb isotope compositions in breastfed newborns resemble those of the mother, as these elements do not undergo significant kinetic fractionation during metabolism. The nutritional composition of milk formula varies between brands, and despite the similarities in composition between cow milk-based infant formulas and human milk, there are notable differences in the nutritional content of formula and breast milk. For example, there are differences in the casein:whey ratio, which is approximately 80:20 in infant formula, and 10:90 in colostrum to 40:60 in mature breast milk. Furthermore, there are variations in the types of casein and whey proteins present, as well as in the percentage of non-protein nitrogen^29^.

To allow for the most accurate identification of geological origin in (newborn) infants, it is of utmost significance to establish the elemental concentrations and isotopic composition among different brands of milk formula. Typically, formula milk is made by mixing industrial milk powder with (tap) water. Although the addition of water has a negligible effect on the C-N-S isotope composition of the resulting milk, it does influence the ^87^Sr/^86^Sr due to the distinct concentrations of strontium and the strontium isotope ratios present in the two components.

Therefore, in this study, the elemental concentration and isotopic composition of Sr-C-N-S of 10 commercially available cow milk formulas (PRE or step 1, 0–6 months) from the Netherlands and Germany are analysed to understand the potential impact of infant formula feeding on the isotopic composition in the body tissues of newborns. Isotopic mixing models are created based on new Sr isotope and concentration data [Sr] from tap water from Germany and previously published data from the Netherlands^19^. Ultimately, the study aims to determine whether isotopic analysis in forensic investigations could be beneficial in cases related to deceased infants.

## Results

The results are presented in Tables 1 and 2. The ^87^Sr/^86^Sr of the baby formula range from 0.70900 (Kruidvat) to 0.71248 (Hero Baby), the [Sr] between 4.35 (Nutrilon) and 6.29 ppm (Babylove). The Munich tap water sample has a ^87^Sr/^86^Sr of 0.70803, and a [Sr] of 0.206 ppm (ng/mg^−1^); relatively low compared to that of the cow milk formulae.

**Table 1:** Sr concentration and isotope data of industrial milk formula brands for newborns from Germany (DE) and the Netherlands (NL) and tap waters.

		[Sr] (ppm)	^87^Sr/^86^Sr	2SE
Formula	Hipp Bio	5.54	0.709275	0.000007
Germany	Milasan	5.69	0.709152	0.000007
	Aptamil	5.39	0.709305	0.000006
	Babydream	5.41	0.709556	0.000007
	Babylove	6.29	0.709268	0.000008
	Bebivita	5.07	0.709283	0.000008
Mean		5.57		
Formula	Albert Heijn	5.20	0.709144	0.000007
The Netherlands	Kruidvat	5.02	0.708998	0.000006
	Nutrilon	4.35	0.709752	0.000008
	Hero Baby	5.41	0.712479	0.000008
Mean		5.00		
Tap water	Munich (DE)	0.21	0.708029	0.000008
	Velp (NL)*	0.04	0.709224	0.000006
	's-Gravenhage (NL)*	0.22	0.709066	0.000008
	Ouddorp (NL)*	0.36	0.708782	0.000007
	Susteren (NL)*	0.13	0.710720	0.000007
Mean		0.19		

Data from tap waters marked with an * are taken from Kootker et al.^19^.

*2SE* two standard error.

**Table 2:** *δ*^13^C, *δ*^15^N and *δ*^34^S (in ‰) of ten industrial milk formula brands for newborns from Germany and the Netherlands.

Formula	*δ*^13^C (‰)					*δ*^15^N (‰)			*δ*^34^S (‰)	
	Bulk	Casein	Whey	Lactose	Lipid	Bulk	Casein	Whey	Casein	Whey
Hipp Bio	− 26.9	− 27.2	− 25.1	− 24.9	− 28.0	3.7	4.5	4.7	4.3	5.0
Milasan	− 24.6	− 24.9	− 21.6	− 22.1	-	4.3	5.0	5.4	4.6	4.5
Aptamil	− 25.5	− 26.4	− 22.7	− 23.6	− 27.3	4.7	5.5	5.6	4.9	4.9
Babydream	− 27.3	− 28.2	− 26.8	− 25.8	-	5.1	6.1	6.4	4.5	5.8
Babylove	− 26.5	− 28.1	− 25.4	− 24.5	-	5.2	6.0	6.4	5.7	7.8
Bebivita	− 25.4	− 27.9	− 22.3	− 22.6	-	4.8	5.7	5.4	4.8	5.5
Mean German brands	− 26.0	− 27.1	− 24.0	− 23.9	− 27.7	4.6	5.5	5.7	4.8	5.6
Albert Heijn	− 25.6	− 25.7	− 22.9	− 24.4	− 27.9	5.1	5.7	5.7	4.1	4.6
Kruidvat	− 25.2	− 25.3	− 24.7	− 22.6	− 29.8	5.5	6.1	6.0	4.5	4.5
Nutrilon	− 25.4	− 24.9	− 22.8	− 23.0	− 28.9	5.8	5.9	5.7	4.7	4.4
Hero Baby	− 25.6	− 26.6	− 23.3	− 23.8	− 28.7	5.0	5.2	5.6	5.3	5.4
Mean Dutch brands	− 25.5	− 25.6	− 23.4	− 23.4	− 28.8	5.4	5.7	5.8	4.6	4.7
Grand mean	− 25.8	− 26.5	− 23.7	− 23.7	− 28.4	4.9	5.6	5.7	4.7	5.2
Standard deviation (1 s)	0.8	1.3	1.6	1.2	0.9	0.6	0.5	0.5	0.5	1.0

The C-N-S isotope values are variable, but the variation is considerably less in the Dutch formula brands (Supplementary data). The *δ*^13^C_bulk_ data vary between − 27.3‰ (Babydream) and − 24.6‰ (Milasan). All Dutch brands exhibit intermediate *δ*^13^C_bulk_ values with limited variation, ranging from − 25.6‰ (Albert Heijn) to − 25.2‰ (Kruidvat). A similar trend is observed in the *δ*^15^N_bulk_ data, with greater variations in the German brands (3.7 to 5.2‰) compared to the Dutch brands (5.0 to 5.8‰). The *δ*^34^S_casein_ data are comparable and show similar variation both the German and Dutch brands (1.4 and 1.2‰ respectively). In contrast, larger variations (3.3 and 1.0‰ respectively) are observed in the *δ*^34^S_whey_ data. In general, all German brands exhibit significant wider ranges in C-N-S isotope data (x̅ = 2.5‰) compared to the Dutch brands (x̅ = 1.2‰).

## Discussion

Variability in the ^87^Sr/^86^Sr of milk formulas was anticipated due to the diverse geographical origins of their constituent raw materials across Europe. Nevertheless, the high ^87^Sr/^86^Sr observed in Hero Baby formula was noteworthy. Upon enquiry, it was disclosed that Hero Baby (now rebranded as Hero Nutrasense) formula is manufactured in Sweden. This accounts for the elevated radiogenic strontium ratios identified in this study due to the ancient regional geology^30^. In manufacture, Hero utilizes multiple herds, albeit all originating from Sweden. If the milk from other companies also comes from multiple farms, this could potentially result in variation in ^87^Sr/^86^Sr between different product batches.

**Figure 1:**
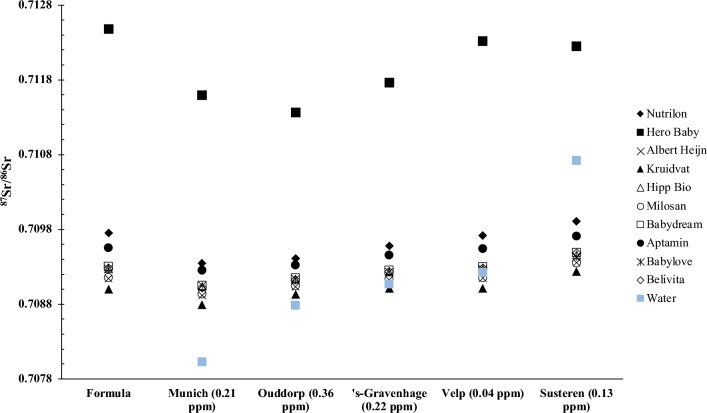
^87^Sr/^86^Sr of the milk formula, the tap water samples, and of the resultant milk. The two standard error (2SE) bars are smaller than the symbols used.

To calculate the Sr isotope composition of a single bottle of formula milk (comprising 30 ml tap water and 1 scoop of formula, totalling 4.6 g), an isotope mixing equation (Eq. 1) was employed. This equation incorporated previously published ^87^Sr/^86^Sr for tap water from the Netherlands and newly generated data for Munich tap water (Table 2). The resulting ^87^Sr/^86^Sr of the formula milk for all brands, in conjunction with the five tap water sources, are tabulated in Table 3 and depicted in Fig. 1.

**Table 3:** Calculated ^87^Sr/^86^Sr of resulting milk using the isotope mixing Eq. 1.

Formula	Tap water sources					Max–min
	Munich	Velp	's-Gravenhage	Ouddorp	Susteren	
Hipp Bio	0.709031	0.709272	0.709232	0.709129	0.709461	0.000429
Milasan	0.708937	0.709155	0.709135	0.709044	0.709349	0.000412
Aptamil	0.709250	0.709539	0.709452	0.709322	0.709709	0.000459
Babydream	0.709051	0.709301	0.709255	0.709148	0.709491	0.000440
Babylove	0.709050	0.709266	0.709230	0.709136	0.709435	0.000386
Bebivita	0.709020	0.709279	0.709235	0.709125	0.709482	0.000463
Albert Heijn	0.708914	0.709148	0.709127	0.709032	0.709358	0.000444
Kruidvat	0.708793	0.709011	0.709014	0.708930	0.709240	0.000446
Nutrilon	0.709345	0.709719	0.709581	0.709413	0.709905	0.000561
Hero Baby	0.711593	0.712315	0.711761	0.711366	0.712248	0.000882
Water	0.708029	0.709224	0.709066	0.708782	0.710720	0.002691

The variation in ^87^Sr/^86^Sr of the resultant milk was relatively minor when compared to the raw milk formulas ^87^Sr/^86^Sr (Table 1). These variations ranged from 0.0000 (Velp tap water + Hipp bio, for example) to 0.0011 (Ouddorp tap water + Hero Baby). In contrast, significantly larger disparities were observed between the ^87^Sr/^86^Sr of the resulting milk and the respective tap water sources, ranging from 0.0000 (Velp tap water + Hipp bio, for instance) to as high as 0.0036 (Munich tap water + Hero Baby).

The minimal difference in Sr isotopic composition between the generated milk and the raw materials can be attributed to the high Sr concentration in milk formula. The average Sr concentration in cow's milk formula (x̅ = 5.3 ppm) is, on average, 15 to 120 times higher than that in the tap water used in this study (x̅ = 0.191 ppm). Consequently, the ^87^Sr/^86^Sr of the formula strongly determines that of the resulting milk.

For this research, the lead (Pb) isotope composition was not determined. However, the concentration measurements showed a 3 to c. 8000 times higher abundance of lead in the formulae (0.012–3.25 ppm) compared to the various tap waters (0.000–0.004 ppm: Supplementary Data). Although this is a significantly lower abundance compared to the observed Sr concentrations (4.35–6.29 ppm and 0.04–0.36 ppm respectively), it is significant enough to conclude that, similar to the Sr isotope system, the Pb of the formula will dominate a baby's bone isotope signatures very quickly.

The implications of these findings are profound for the application of the strontium isotope system in forensic investigations, particularly in cases involving very young infants. To successfully use Sr isotope data to geolocate the deceased infant’s mother, it is essential to determine age-at-death, and the primary food source at and prior to death accurately and precisely. In situations involving neonates who passed away before, during, or shortly after birth, the ^87^Sr/^86^Sr in bone and hair tissues will mirror those of the biological mother. However, in older individuals fed with formula milk for extended periods, spanning weeks or even months, the ^87^Sr/^86^Sr in their bones may be partially overwritten by the ^87^Sr/^86^Sr of the formula due to the rapid turnover rate of bones. Consequently, after a few weeks, the ^87^Sr/^86^Sr in their tissues will no longer accurately represent the geological origin of the mother but instead reflect the composition of the formula milk used. Therefore, it is crucial for the interpretation of the Sr isotope data from newborns to determine the main source of milk after birth. In this context the stable isotope data are vital.

Previous studies reported isotopic enrichments of 1–2‰ for *δ*^13^C and 2–3‰ for *δ*^15^N in breastfed infant's keratinous tissues compared to maternal keratinous tissue values, and a difference of approximately 4–5‰ for *δ*^13^C and 3–5‰ for *δ*^15^N between the isotopic composition of milk (maternal and formula) and that of the infant’s keratinous tissues up to c. 4 weeks after birth^26,28^. It must be noted, however, that the number of reference data are limited, with only one for the maternal—formula milk offset. In addition, differences in enrichment between countries must be expected due to the cultural differences in diet. Moreover, the maximum enrichment of ca. 5–6‰ in carbon and nitrogen will be reached > 4 weeks^31^. Thus, isotopic shifts between infant and mother are expected, but the range of variation is not yet well understood and requires more in-depth research. However, for this study, it is anticipated that young infants in the Netherlands and Germany, exclusively fed with the investigated cow milk formula up to c. 17 weeks when solids foods are introduced, could exhibit *δ*^13^C values of approximately − 21 to − 22‰ and a *δ*^15^N value of around 8 to 10‰ (mean formula *δ*^13^C_bulk_ and *δ*^15^N_bulk_ values plus respectively 4–5‰ and 3–5‰ shifts, mirroring previously published data^26,31^).

**Figure 2:**
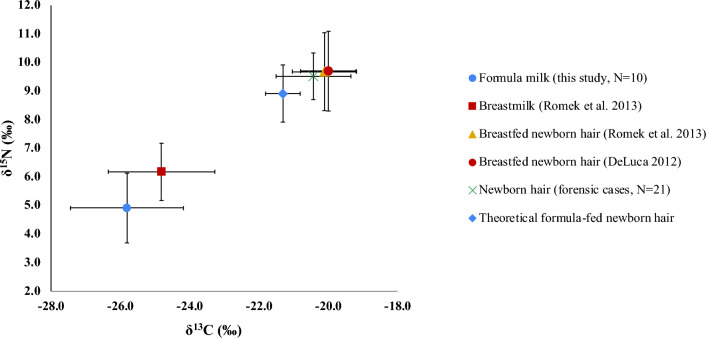
Reference mean ± 2 s carbon and nitrogen isotope values of formula milk (this study) and breastmilk^25,28^, hair from newborns^28^ and newborns from forensic cases (this study), and theoretical data of bottle fed babies with formula milk used in this study.

Figure 2 shows the mean *δ*^13^C and *δ*^15^N values (± 2 s) of previously published breast milk and hair from exclusively breastfed infants (up to 4 weeks after birth), together with the mean ± 2 s bulk data of the Dutch and German milk formula, the isotopic values of hair samples obtained from 25 forensic cases in Germany of which most died immediately after birth without being nourishing with formula or breast milk (mean ± 2 s, with a range from − 21.6 to − 18.8‰ for *δ*^13^C and 8.9‰ to 10.2‰ for *δ*^15^N, respectively), and predicted *δ*^13^C and *δ*^15^N for milk formula fed babies up to c. 4 weeks using a theoretical + 4.5 ± 0.5‰ for *δ*^13^C and + 4.0 ± 1.0‰ for *δ*^15^N shifts^26^.

The carbon and nitrogen isotope values of the forensic hair samples representing the maternal isotope signature exhibit an average enrichment of + 5.4‰, and + 4.9‰ respectively, in comparison to the *δ*^13^C and *δ*^15^N data of the milk formula analysed in this study. Similarly, these values are enriched by + 4.3‰, and + 3.3‰, respectively, when compared to breastmilk data^28^. These values align with previously published data for breastfed newborns during the first weeks after birth. The theoretical *δ*^13^C and *δ*^15^N values of unweaned bottle-fed infants are slightly depleted in comparison to the hair of breastfed infants (− 21.3‰ and − 20.2‰ for *δ*^13^C, and 8.9‰ and 9.7‰ for *δ*^15^N, respectively).

The data presented here show the opportunity for statistical analyses to identify milk formula fed infants, and potentially the type or even geographical origin of the source milk, in larger actualistic datasets. Importantly, determining the maternal *δ*^13^C and *δ*^15^N values could hold significance in forensic investigations related to maternal geolocation, as demonstrated in previous research^32^. If the primary source of milk (maternal *vs*. industrial) cannot be determined, the hair tips of deceased newborns or infants can be targeted for analysis, as they are formed during pregnancy and carry the maternal Sr-C-N-S isotopic composition, provided the baby's hair has not yet undergone shedding (neonatal occipital alopecia, or NOA).

Unfortunately, comparative *δ*^34^S data are currently scarce^33^, as are C-N isotope data from other countries in Europe, further underlining the need for more research on C-N-S isotopic fractionation between formula and breastmilk, and baby tissues.

## Conclusions

This study reports the Sr-C-N-S isotope composition of industrial milk formulas from Germany and the Netherlands. Generally, the *δ*^13^C and *δ*^15^N values of the milk formulas are comparatively depleted relative to those inherent in maternal milk. Consequently, by applying a comparable trophic enrichment observed between the milk samples and newborns' hair, it is anticipated that hair samples from exclusively industrial formula milk fed infants will manifest lower *δ*^13^C, and potentially lower *δ*^15^N compared to infants who are breastfed.

Utilizing an isotope mixing equation incorporating tap water data from the Netherlands and Munich, the study establishes that the relatively high Sr content of milk formula (4.35–6.29 ppm) has a major influence on the ^87^Sr/^86^Sr of milk formula. The implications of these findings in forensic investigations, especially concerning young infants, are significant. The study emphasized the importance of accurately determining age-at-death and primary food source (maternal or formula milk) in forensic cases involving neonates. In cases of extended formula feeding, the ^87^Sr/^86^Sr in hair and bones is expected be influenced by the formula, strongly impacting, even limiting, the applicability of Sr isotope analysis as a tool to geolocate the infant or the birthmother. This necessitates the need to continue building actualistic C-N-S isotope datasets of breastmilk, and (keratinous) tissues from exclusively breastmilk fed babies and milk formula fed babies, to enable the isotopic differentiation between breastfed and formula milk fed babies.

## Material and methods

A total of ten brands of baby formula from Germany (N = 6) and the Netherlands (N = 4) were analysed (Table 1 and Supplementary data). Baby formulas were purchased in 2021. The powder of the German brands was sent in 50 ml vials to the Vrije Universiteit Amsterdam for the analysis of Sr isotopes and 50 ml of Dutch milk powders were sent to the Ludwig-Maximilians-Universität in München for the analysis of C-N-S isotopes.

### Stable isotope analysis

For bulk analysis of C-N-S isotopes, 3 mg of formula were weighted into tin capsules. For lipid carbon isotope analyses lipid was extracted from 1 g formula using petroleum ether. After evaporation, 2 mg were weighted into tin capsules. All samples were analysed in quadruplicate. Casein was precipitated from the lipid-free solution of 5 g of formula dissolved within 10 ml of water by acidification with 0.1 N HCl to pH 4.3 and subsequent centrifugation. The precipitate was rinsed with water and lyophilised. The supernatant and washings were combined and lyophilised. This residue consisted of lactose and whey proteins. To precipitate the whey protein, the dried residue was dissolved in deionized water, heated, and sodium tungstate and 0.1 N H_2_SO_4_ were added. The protein was subsequently separated from the supernatant by centrifugation. The supernatant was then lyophilised to obtain the lactose.

Stable C-N-S isotope ratios of the samples were determined using an Elementar Vario Cube EL instrument (Elementar Analysensysteme GmbH, Hanau, Germany) connected with an Isoprime mass spectrometer (Isoprime Ltd. Cheadle Hulme, UK) or an Elementar Pyrocube connected with an Isoprime VisIon mass spectrometer at Isolab GmbH, Schweitenkirchen, Germany, following established protocols^34^*.* For δ^15^N and δ^34^S, scale calibrations with organic reference materials (collagen) were performed (USGS 88 and 89 for both δ^15^N and δ^34^S)^35^. In addition, a casein standard characterised by the working group ‘Stable Isotope Analytics’ of the Gesellschaft Deutscher Chemiker (GDCh) was used^36^. Scale calibration for *δ*^13^C was performed with NIST SRM 22 and IRMM-BCR 657, in addition to USGS88 and USGS89 collagen and casein samples^37^. All *δ* values were indicated in per mil (‰) relative to international reference standards, *δ*^13^C_VPDB_, *δ*^15^N_VAIR_ and *δ*^34^S_VCDT._ Analytical precisions using measurements in at least triplicate were ± 0.1‰ for *δ*^13^C, ± 0.2‰ for *δ*^15^N, and ± 0.3‰ for *δ*^34^S.

### Radiogenic isotope analysis

Between 218 and 252 mg of formula was weighted into acid-precleaned 7 ml Savillex PFA vials and transferred to the USA class 100 (ISO 5) clean laboratory with USA class 10 laminar flow hoods (ISO 4) at the Vrije Universiteit Amsterdam. Here, 5 ml 3 M HNO_3_, 5 ml 6–7 M HCl and c. 300 µl H_2_O_2_ was added to remove all organic fractions and to completely dissolve the samples in closed beakers at 120 °C. In addition, circa 5 ml of tap water was collected in an acid-precleaned 7 ml Savillex PFA. All samples were dried overnight on a hot plate at 120 °C and subsequently redissolved in 500 µl 3 M HNO_3_. Depending on the sample weight, an aliquot of c. 20 µl was taken and together with ca. 180 µl 5% HNO_3_ collected in acid cleaned ICP Exetainer® vials for the measurement of elemental concentrations. For the Munich water sample, a 2 ml aliquot of the stock tap water sample was pipetted into a 10 ml acid cleaned ICP Exetainer^®^ vial and acidified with 118 µl 14 M HNO_3_ to make a solution of 5% HNO_3_.

Strontium was extracted using standard ion-exchange techniques^38^. After adding one drop of H_3_PO_4_, the samples were dried overnight, nitrated with c. 150 µl 14 M HNO_3_ to remove all organic contents, and dried again.

The samples were then transferred to the mass spectrometry laboratory at the Vrije Universiteit Amsterdam where they were redissolved in 2 µl HNO_3_. One µl was loaded on single Re annealed filaments using 2 µl TaCl. Strontium isotope compositions were measured using a Thermo Scientific™ Triton Plus™ Thermal Ionization Mass Spectrometer (TIMS). The strontium isotope ratios were determined using a static routine with amplifier rotation and corrected for mass fractionation to ^86^Sr/^88^Sr ratio of 0.1194. The long-term reproducibility in 2021 was 0.710255 ± 0.000017 for repeated analysis of the NIST SRM 987 standard (N = 156, 2 s, loading size 200 ng). The total procedural blank contained a negligible amount of Sr. The ^87^Sr/^86^Sr are reported plus minus two standard errors (2SE), representing the analytical uncertainty calculated from maximum 240 measurements (12 blocks of 20 cycles) within each run.

### Concentration measurements

Strontium concentrations were measured on a Thermo X-Series II Inductively Coupled Plasma Mass Spectrometer (ICP-MS). The aliquot solutions were introduced through a quartz dual cyclonic spray chamber equipped with a PFA-ST MicroFlow nebuliser (Elemental Scientific) with a sample uptake rate of about 100 µl min^−1^. Due to a lack of matrix-matched reference materials at the Vrije Universiteit Amsterdam at the time of the study, geological reference materials were used for calibration, sensitivity-drift correction, and quality control. The reference materials AGV-2, G-2 and RGM-2 were used as calibrants, in addition to an acid blank. The reference material BHVO-2 was used as a quality-control standard to evaluate accuracy. The average bias (the relative deviation from the GeoReM preferred value of 394.1 ppm) of Sr concentrations determined for BHVO-2 were 3, 2, and 11% for concentrations determined from ^84^Sr, ^86^Sr and ^88^Sr, respectively. Although using geological reference materials for the water and formula samples is non-ideal, the following measures were taken: 1) the geological reference materials were diluted ~ 5000 times to minimize high-matrix effects, and 2) the samples were diluted such that the Sr signals were within the same approximate range as the geologic reference materials. The Sr isotope ratios for most samples were measured in the same detector mode (pulse or analog) as the reference materials for the corresponding isotope. Although uncertainties on the Sr concentrations are difficult to estimate due to lack of matrix-matched standards, reasonable uncertainties do not have an effect on the conclusions of the mixing models.

### Mixing model analysis

A reworked mixing equation from Faure and Mensing was applied to calculate the Sr isotope composition of the resulting milk formula (Eq. 1)^39^.

1 $${\left[\frac{{}^{87}Sr}{{}^{86}Sr}\right]}_{MIX }= \frac{{X}_{A}{\left[Sr\right]}_{A }{\left(\frac{{}^{87}Sr}{{}^{86}Sr}\right)}_{A}+{{X}_{B}\left[Sr\right]}_{B }{\left(\frac{{}^{87}Sr}{{}^{86}Sr}\right)}_{B} }{{X}_{A}{\left[Sr\right]}_{A }+{X}_{B}{\left[Sr\right]}_{B}}$$

X_A_ and X_B_ are the weight fractions of components A (formula) and B (water), [Sr]_A_ and [Sr]_B_ are the abundances of Sr, and (^87^Sr/^86^Sr)_A_ and (^87^Sr/^86^Sr)_B_ are the isotopic ratios of the formula and water respectively.

### Supplementary Information

Supplementary Information.

## Data Availability

All data generated during this study are included in this published article.
