# Supplementary material for: Isotopic analysis of formula milk reveals potential challenges in geolocating bottle-fed babies
Source: Sci Rep. 2024 Feb 13;14:3600. doi: 10.1038/s41598-024-54173-y (PMC11341844; doi:10.1038/s41598-024-54173-y)
Supplement: Supplementary file 1 — Supplementary Information. [file 41598_2024_54173_MOESM1_ESM.pdf]

Supplementary data

Isotopic Analysis of Formula Milk Reveals Potential Challenges in Geolocating Bottle-fed Babies

Lisette M. Kootker1,2*, Saskia T.M. Ammer1,2, Gareth R. Davies1,2, Christine Lehn3

1 Vrije Universiteit Amsterdam, Faculty of Science, Department of Earth Sciences, Geology & Geochemistry cluster, de Boelelaan 1085, 1081 HV Amsterdam, the Netherlands
2 Co van Ledden Hulsebosch Center (CLHC), Science Park 904, 1098 XH Amsterdam, the Netherlands
3 Ludwig-Maximilians-Universität München, Department of Forensic Medicine, Nußbaumstraße 26,
80336 Munich, Germany

		in ppm				
**Sample type**	**VU Code**	**[Sr]**	**[Pb]**	**Bought/collected in**	**Samples**	
Formula	E216	5.542	0.0184	Germany	Hipp Bio	
Formula	E217	5.196	0.0146	the Netherlands	Albert Heijn	
Formula	E218	5.020	0.0116	the Netherlands	Kruidvat	
Formula	E219	5.693	0.0191	Germany	Milosan	
Formula	E220	4.350	0.0124	the Netherlands	Nutrilon	
Formula	E221	5.411	0.0147	the Netherlands	Hero Baby	
Formula	E222	5.412	3.2450	Germany	Babydream	Above WHO threshold of 20 ppb or 0.02 ppm
Formula	E223	5.387	0.0131	Germany	Aptamil	
Formula	E224	6.289	0.0154	Germany	Babylove	
Formula	E225	5.075	1.4360	Germany	Bebivita	Above WHO threshold of 20 ppb or 0.02 ppm
Tap water	E278	0.206	0.0032	Germany	Munich	
Tap water	Z887	0.044	0.0008	the Netherlands	Velp	
Tap water	Z886	0.221	0.0039	the Netherlands	's-Gravenhage	
Tap water	Z922	0.357	0.0004	the Netherlands	Ouddorp	
Tap water	B035	0.126	0.0016	the Netherlands	Susteren	

Formula	MIN	4.350	0.0116	
	MAX	6.289	3.2450	
Tap water	MIN	0.044	0.0004	
	MAX	0.357	0.0039	
		**MIN**	**3.0**	**times as much Pb in formula than in tap water**
		**MAX**	**8113**	**times as much Pb in formula than in tap water**
